# Ultrasound Imaging for Risk Assessment in Atherosclerosis

**DOI:** 10.3390/ijms16059749

**Published:** 2015-04-29

**Authors:** David C. Steinl, Beat A. Kaufmann

**Affiliations:** 1 Department of Biomedicine, University Hospital Basel, Hebelstrasse 20, Basel 4031, Switzerland; E-Mail: david.steinl@unibas.ch; 2 Division of Cardiology, University Hospital Basel, Petersgraben 4, Basel 4031, Switzerland

**Keywords:** ultrasound, contrast enhanced, molecular imaging, atherosclerosis, micro-bubbles

## Abstract

Atherosclerosis and its consequences like acute myocardial infarction or stroke are highly prevalent in western countries, and the incidence of atherosclerosis is rapidly rising in developing countries. Atherosclerosis is a disease that progresses silently over several decades before it results in the aforementioned clinical consequences. Therefore, there is a clinical need for imaging methods to detect the early stages of atherosclerosis and to better risk stratify patients. In this review, we will discuss how ultrasound imaging can contribute to the detection and risk stratification of atherosclerosis by (a) detecting advanced and early plaques; (b) evaluating the biomechanical consequences of atherosclerosis in the vessel wall; (c) assessing plaque neovascularization and (d) imaging the expression of disease-relevant molecules using molecular imaging.

## 1. Introduction

Atherosclerosis is a systemic, multifactorial disease affecting large arteries throughout the body [1]. The disease process is mainly driven by two underlying processes: a disturbed equilibrium of lipid accumulation and chronic inflammation of the vessel wall [2], which together leads to the buildup of atherosclerotic plaques that, in turn, can lead to a variety of cardiovascular diseases and complications.

Autopsy studies have shown that the pathogenesis of atherosclerosis starts at a young age [3,4] and thus can progress silently over decades before it results in life-threatening vascular complications (acute myocardial infarction, cerebrovascular insult). Death statistics in Europe show that nearly every second death is attributable to cardiovascular diseases [5]. In the last four decades, however, rates of death from cardiovascular disease have declined in the western world, whereas disease prevalence has increased in developing countries, and it is estimated that today, 80% of the global burden of cardiovascular disease is occurring in these countries [6].

Given the natural history of atherosclerosis with a silent progression over several decades, reliable methods for risk estimations in individuals are a clinical need. Current approaches to risk stratification rely on well-established clinical risk scores (Framingham, PROCAM (Prospective Cardiovascular Münster Study), ESC-SCORE (European Society of Cardiology-Systematic Coronary Risk Evaluation)) that incorporate clinical risk factors for atherosclerosis (arterial hypertension, diabetes mellitus, hypercholesterolemia, smoking, and family history for premature coronary artery disease) [7]. However, when applying these risk scores to populations in western countries, approximately 40% of the adult population fall into a medium risk category, in which the benefit of a broad use of preventive strategies is uncertain. Therefore, further risk stratification in this group for the allocation of preventive therapies and/or strategies is desirable. In addition, those methods should optimally allow for the subsequent assessment of therapy effects aimed at inhibiting the progression of atherosclerosis.

In the last decades, developments in all major non-invasive imaging technologies have been used to assess the atherosclerotic disease process. When envisaging application of such an imaging method in risk prediction, ultrasound has distinct advantages over other imaging modalities in terms of wide availability and low cost.

In this review, we will first give an overview of the vascular biology of the pathogenesis of atherosclerosis. We will then review how conventional ultrasound imaging can be used to assess arteries for the presence of atherosclerosis, and what complex signal processing algorithms can possibly add to the evaluation of conventional ultrasound images. We will then explain the basic principles of molecular imaging using targeted contrast agents, and will review how this novel methodology has been used in relevant animal models of disease to detect the presence and response to therapy of vascular inflammation that drives the pathogenesis of atherosclerosis.

## 2. Atherosclerosis

**Figure 1:**
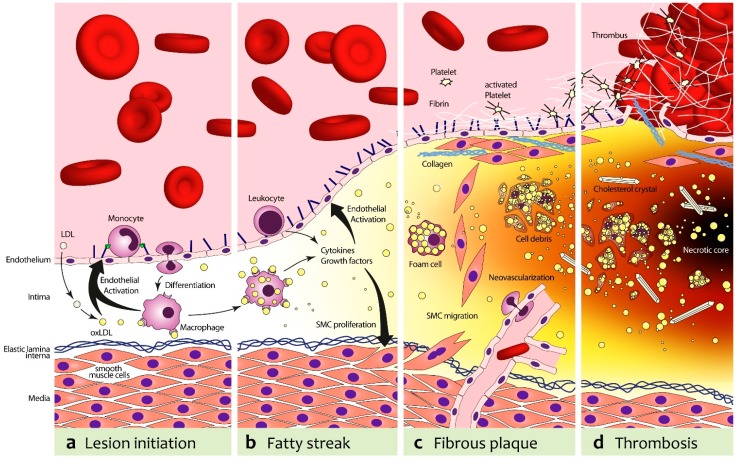
Pathogenesis of atherosclerosis. (**a**) In the first stage, low density lipoprotein-cholesterol (LDL) is deposited in the endothelium and undergoes oxidative modification, resulting in oxidized LDL (oxLDL). OxLDL stimulates endothelial cells to express adhesion molecules (vascular cell adhesion molecule-1 (VCAM-1), P-Selectin) and various chemokines (e.g., Monocyte Chemoattractant Protein-1 (MCP-1), Interleukin 8 (IL-8)). This leads to a recruitment of monocytes, which transmigrate into the intima and differentiate to pro-atherogenic macrophages; (**b**) Macrophages harvest residual oxLDL via their scavenger receptors and add to the endothelial activation and, subsequently, leukocyte recruitment with the secretion of Tumor Necrosis Factor α (TNF-α) and IL-6; (**c**) The increasing plaque volume promotes neovascularization. Proliferating smooth muscle cells (SMCs) stabilize the nascent fibrous plaque. With deposition of fibrin and activated platelets on the dysfunctional endothelium that expresses tissue factor (TF) and von Willebrand factor (vWF), a pro-thrombotic milieu is formed; (**d**) Foam cells can undergo apoptosis and release cell-debris and lipids, which will result in the formation of a necrotic core. In addition, proteases secreted from foam cells can destabilize the plaque. This can lead to plaque rupture, in which case extracellular matrix molecules (e.g., collagens, elastin, TF, vWF) catalyze thrombotic events.

While incompletely understood, the first step in the development of vascular endothelial inflammation that leads to the development of atherosclerotic plaques, seems to be sub-endothelial deposition of low density lipoprotein-cholesterol (LDL). LDL is subsequently modified to oxidized LDL (oxLDL), a process that is influenced by cardiovascular risk factors such as hypertension, diabetes, or smoking that all increase oxidative stress in the vascular wall [8,9,10]. Through activation of NF-κB, oxLDL leads to the expression of vascular cell adhesion molecule-1 (VCAM-1) and intercellular adhesion molecule-1 (ICAM-1) on the vascular surface of endothelial cells [11]. By interaction with their counter-ligands α4:β1 and CD11a:CD18 on monocytes [12], the expression of these cell adhesion molecules leads to recruitment of monocytes into the vascular wall [13] (Figure 1).

Once in the vascular wall, the local inflammatory milieu further activates the monocytes, which are converted to macrophages that scavenge oxLDL and act to amplify the inflammatory process via the secretion of cytokines (IL-1β, IL-12, TNF-α) [14]. Continuing recruitment of monocytes leads to intimal thickening and the formation of fatty streaks, which are the earliest lesions of atherosclerosis that can be appreciated macroscopically. Continuing inflammatory cell recruitment leads to plaque growth and recruitment of additional cell types (vascular smooth muscle cells, VSMC). Plaque growth also leads to local tissue hypoxia, which promotes neovascularization of the atherosclerotic lesions [15]. The inflammatory activation of the endothelium not only leads to recruitment of leukocytes, but via the expression of von Willebrand factor (vWF) [16] and tissue factor (TF) [17] also creates a pro-thrombotic environment. Intimal thickening, plaque formation, plaque neovascularization, and the expression of cell adhesion molecules and pro-thrombotic factors are all potential targets for imaging of atherosclerosis with ultrasound.

The structural changes in the arterial wall during the pathogenesis of atherosclerosis can be conceptualized as vascular aging, the pace of which is correlated to the risk for cardiovascular events in individuals [18]. These morphological and functional changes are a consequence of the underlying molecular pathways described above, and include changes of the general vessel morphology with buildup of plaque [19], alterations in the biomechanical properties of the arterial wall [20], and the development of atherosclerotic plaque neo-vascularization [21]. These parameters can be assessed by different non-invasive imaging techniques, usually performed on either the carotid arteries or the aortic arch, the changes of which are accepted surrogate markers for atherosclerotic disease progression in the coronaries and cardiovascular events [22].

## 3. Anatomical Imaging of Atherosclerosis with Ultrasound

**Figure 2:**
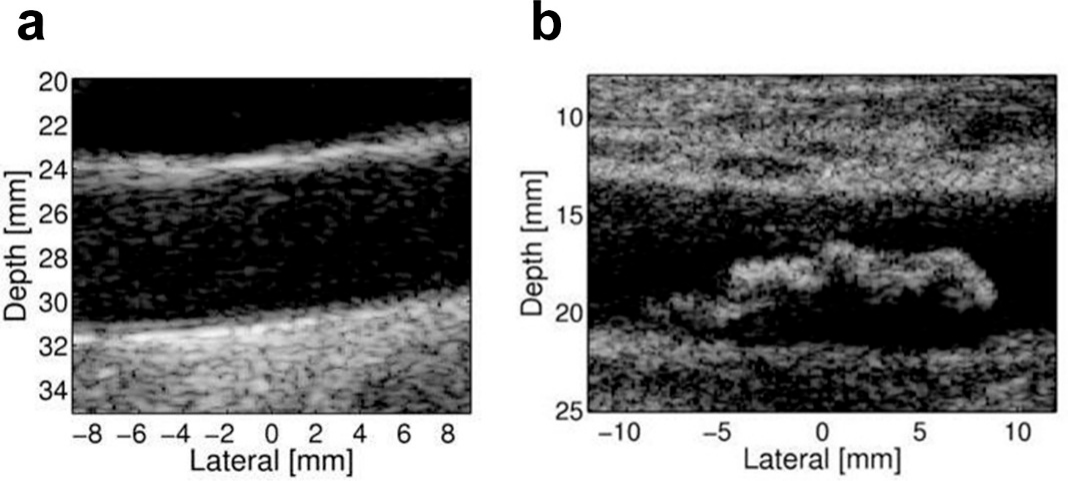
B-mode imaging of the carotid artery. These images illustrate (**a**) a normal carotid artery; and (**b**) a large atherosclerotic plaque protruding into the lumen of the carotid artery. Reproduced from [25], with permission.

Established atherosclerotic lesions can be visualized with anatomical B-mode ultrasound imaging as protrusions of the intima-media (Figure 2) and the number of visualized plaques, as well as total plaque area or total plaque volume was reported to be an independent predictor of future cardiovascular mortality and coronary events [23,24].

Additional information can be derived from plaque echogenicity. On B-mode ultrasound, plaques that contain a large lipid core appear echolucent, whereas plaque fibrosis and calcification tend to appear echogenic. As proposed by Gray-Weale *et al.* [26], carotid plaques can be classified into four categories as echolucent, predominantly echolucent, predominantly echogenic, or echogenic. In patients with carotid stenosis, the degree of plaque echolucency correlated with increased risk for cerebrovascular events [27]. Similarly, standardized measurements of the decrease in gray scale levels within carotid plaques over time have been correlated to the risk of subsequent cardiovascular events [28].

Plaques that are large enough to be visualized with ultrasound develop relatively late in the pathogenesis of atherosclerosis. However, increases in the carotid intima media thickness (C-IMT), which occur prior to plaque development, can be measured with high-resolution ultrasound. An increase of C-IMT has been found to be associated with risk for cardiovascular events in several large observational studies [29,30]. Thus, C-IMT measurements have a value in population studies, and may even be useful for initial evaluations of the effect of new therapies targeting atherosclerosis before the start of large-scale morbidity and mortality trials [31]. However, a recent meta-analysis has cast doubt on the value of C-IMT for risk prediction in individual patients, showing little added benefit over traditional risk assessment using the Framingham Score with small net reclassification improvements in risk category that are unlikely to be clinically relevant [32]. There are several possible reasons for this lack of additional prognostic information. C-IMT is frequently measured in the common carotid artery, whereas advanced lesions prone to complications develop in the bulb or proximal internal carotid artery. While C-IMT correlates to age and blood pressure, it also offers little advantage over these traditional risk factors for predicting events [33]. Another reason may be that the differences in C-IMT between risk strata is around 200 µm, which is below the axial resolution of ultrasound systems commonly used for these measurements. Algorithms that help measuring C-IMT more precisely [34] or the use of three-dimensional ultrasound that would allow volumetric assessment of plaque burden [35] might potentially help to improve diagnostic value of C-IMT. In this respect, a recent study that used two-dimensional short axis images of the carotid arteries to assess a global, three-dimensional plaque burden of both carotids has shown incremental predictive value over traditional risk factors, which was, in addition, comparable to coronary artery calcium scoring [36].

Catheters in the millimeter size domain offer the possibility for intravascular ultrasound (IVUS) to gain information from inside the vessel in return for sacrificing non-invasiveness. As opposed to angiography, which shows only plaques that lead to a coronary stenosis, IVUS is able to also detect plaques with an eccentric remodeling that do not narrow the vessel lumen. This is important, since eccentric plaque remodeling does not preclude these plaques from causing complications such as plaque rupture, which can lead to vessel occlusion and myocardial infarction [37].

The atheroma burden measured with grayscale IVUS correlates closely with histology findings [38,39,40,41]. It has been shown that when patients with known coronary artery disease are followed up after examination with IVUS, the majority of acute coronary syndromes are due to complications at sites in the coronary tree, which had exhibited an eccentric plaque without stenosis [37].

Gray-scale IVUS does reveal some information about the composition of atherosclerotic lesions and plaques can be classified based on their visual appearance as echolucent (“soft”), hypoechogenic (fibrous), hyperechogenic with or without shadowing (calcified), or intermediate form lesions [42]. However, with gray-scale IVUS calcified plaques cannot be discriminated from densely fibromuscular tissue, both of them hyperechogenic, while lipid-rich plaques cannot be discriminated from fibrotic plaques or intraplaque hemorrhages that appear hypoechogenic to echolucent. Thus, it does not come as a surprise that, in clinical studies, IVUS has not been capable to discriminate between plaques found in patients with stable angina and unstable atherosclerotic lesions [43].

## 4. Biomechanical Imaging

In the evaluation of atherosclerosis, valuable information can not only be obtained from wall morphology or plaque composition but can also be derived from the arterial wall mechanical properties, which change along with the progression of atherosclerosis [44]. Fibrosis, calcification, and increased smooth muscle cell proliferation change the biomechanical properties of the arterial walls, which translate into an increase in Young’s elastic modulus and changes of other parameters of vessel wall deformability or stiffness.

Because in less elastic arteries the speed of pulse wave propagation is increased due to a diminished “Windkessel” capacity, measurements of arterial pulse wave velocity (PWV) with transoesophageal echo (TOE) or transthoracic echo (TTE) doppler ultrasound imaging have been shown to be an independent predictor of atheroma burden and cardiovascular events [45]. However, while a strong association of changes in PWV with age and blood pressure has consistently been shown, the association of these changes with other risk factors for atherosclerosis is unclear [46].

While non-invasively measured PWV is used to assesses changes in the mechanical properties of long segments of arteries and thus, in overall risk prediction, invasive techniques have also been developed in an effort to match changes in local biomechanical properties to plaque composition and, thus, to the risk for vascular complications. Elastography, which derives strain from high-frequency IVUS for a set intravascular pressure differential has been shown to identify plaque components *in vitro* in excised human arteries [47], with fatty, potentially unstable plaques exhibiting a higher deformability and higher strain values. Similar techniques are used to assess vessel strain with palpography, however, in contrast to elastography, only the first 450 µm starting from the luminal side of the vessel are examined. Because the blood pressure, which is the force leading to deformation of the vessel wall, is primarily acting on the luminal side of the vessel wall, this technique is regarded as being more robust for differentiating between different plaque components compared to elastography. [48]. Palpography has been validated *in vivo* in an animal model of atherosclerosis [49] and was able to differentiated plaques with increased fatty or macrophage content from other, more stable plaques. In a study using three-dimensional palpography in patients either with stable angina, unstable angina or acute myocardial infarction, the number of highly deformable plaques on plapography correlated with the clinical presentation [50].

For the non-invasive assessment of vessel elasticity, high frame rate ultrasound imaging [51], velocity vector imaging [52], or strain imaging of the carotid arteries as a reference vessel for cardiovascular risk have been used. Speckle tracking strain imaging of carotid artery plaques has been validated against sonomicrometry [53] and preliminary studies in humans showed an association of carotid artery circumferential strain derived using speckle tracking with previous strokes [54].

## 5. Contrast Enhanced Ultrasound Imaging of Atherosclerosis

Ultrasound contrast agents were developed in the early 1990s with the goal of contrasting the blood pool to allow for better delineation of the left ventricular endocardial border. The use of microbubbles for this purpose is based on observations made by Gramiak and Shah, who noticed a signal enhancement of blood in the aorta after injection of mechanically agitated saline containing small air bubbles [55]. These air bubbles were very short lived and also not suitable for venous injection. Later, ultrasound contrast agents for intravenous injection were developed. Commercially available and FDA-certified are microbubble-based contrast agents, which have a gaseous core encapsulated with a thin shell. To increase microbubble stability, large-molecule, hydrophobic gases such as sulfur hexafluoride or perfluorocarbons are used. For encapsulation, the microbubble shell is composed of combinations of albumin, galactose, lipids, or polymers. These microbubbles measure on average 1–2 µm in diameter and thus circulate freely throughout the microcirculation [56]. In addition to microbubbles, nanoscale echogenic liposomes have also been used as ultrasound contrast agents [57].

### 5.1. Physical Aspects of Contrast Enhanced Ultrasound Imaging

Microbubbles undergo volumetric oscillations with compression during the pressure peaks, and expansion during the pressure nadirs of an incident ultrasound wave [58,59]. These volumetric oscillations result in a strong backscattered acoustic signal that can be detected by ultrasound systems.

The oscillation of a microbubble depends on the frequency and the intensity of an incident sound wave [60]. With low transmitted powers, microbubbles will oscillate at the same frequency as the incident sound wave and thus will backscatter sound waves at the same frequency (linear backscatter). At medium transmitted powers, microbubbles show non-linear oscillations and will thus backscatter sound waves at both the incident frequency and multiples of the incident frequency (harmonic frequencies). As tissue shows a lesser degree of nonlinear behavior, this phenomenon can be exploited to improve the contrast to tissue signal ratio. At high-transmitted powers, the microbubble shell will be disrupted and the microbubble will be destroyed.

### 5.2. Plaque Neovascularization

Healthy arteries exhibit a vascularization of the outer adventitial wall layers but not of the intima and inner media, which are supplied with nutrients by the luminal blood flow. However, with a thickening of the arterial wall as a consequence of the buildup of atherosclerotic plaque, tissue hypoxia develops and stimulates sprouting of the Vasa vasorum into the medial and intimal layers of plaques [61], resulting in plaque neovascularization. Neovessels that develop in large plaques lack pericytes and are prone to rupture [62], which may lead to acute atherosclerotic complications. Plaque neovessels have been shown to promote plaque progression through deposition of cholesterol and macrophages and enlargement of the plaque necrotic core [63]. Thus, it is thought that the detection of plaque neovascularization could be used as a marker for the risk for subsequent cardiovascular events.

**Figure 3:**
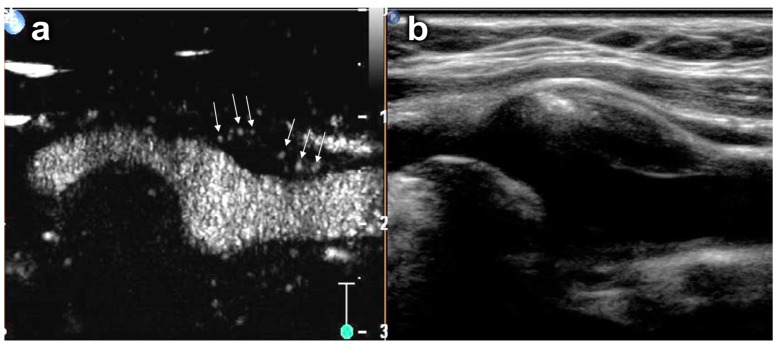
Contrast-enhanced ultrasound (CEUS) imaging of plaque neovascularization in a carotid plaque. (**a**) Carotid artery with intraplaque neovascularization on CEUS. The arrows denote microbubbles within neovessels in a plaque at the origin of the internal carotid artery; (**b**) Corresponding B-mode ultrasound image. Reproduced from [71] with permission.

Contrast enhanced ultrasound imaging has been used to detect neovascularization in carotid plaques (Figure 3). Initial studies have shown that qualitative assessments of plaque neovascularization, as detected with contrast-enhanced ultrasound, correlate to histologically determined micro-vascular density and to plaque echolucency, the latter being a marker of plaque vulnerability [64,65]. Similarly, quantitative data on plaque neovascularization obtained using maximum intensity projection methods have been shown to correlate with histologic neovascularization and inflammatory cell infiltration [66]. A retrospective analysis has shown a correlation of the degree of plaque neovascularization as assessed with contrast-enhanced ultrasound with previous cerebrovascular and coronary events [67]. Interestingly, the distribution of neovascularization within carotid plaques offers additional information on plaque vulnerability with a higher frequency of neovascularization predominantly of the shoulder regions in symptomatic patients [68]. However, prospective studies that assess the predictive value of this method to estimate the risk of future cerebrovascular events have not been published as of to date. Additionally, contrast imaging of the carotid arteries suffers from a pseudoenhancement artefact adjacent to the far wall of the artery that could potentially hamper assessment of plaque neovascularisation in this area [69]. However, novel imaging algorithms using detection methods based on counterpropagation of ultrasound pulses have been described that can potentially eliminate this artefact [70].

### 5.3. Basic Principles of Molecular Imaging with Ultrasound Contrast Agents

The nonlinear signal generation of freely circulating microbubbles is used in clinical practice for opacification of the blood pool or for the assessment of organ perfusion. However, the goal of molecular imaging is the specific attachment of these microbubbles to a relevant target of interest. An ultrasound image of these retained microbubbles should then represent the level of expression of the target in the tissue. The target-specific retention of microbubbles can be accomplished by either (1) modifications of the microbubble shell components that facilitate attachment of microbubbles to activated leukocytes; or (2) the conjugation of ligands specific for a disease molecule to the microbubble surface. An example for microbubble targeting using modified shell characteristics is the incorporation of negatively charged phospholipids into the microbubble shell. The negative microbubble surface charge leads to complement deposition on its surface, which, in turn, promotes attachment to activated leukocytes [72]. Similarly, microbubbles with an albumin shell have been shown to bind to monocytes and neutrophils through attachment to the leucocyte β2-integrin Mac-1 (CD11b/CD18) [73]. Much more versatile is the strategy of coupling ligands like antibodies, glycoproteins, carbohydrates or peptides to the microbubble surface. Coupling of the ligands is done either directly to the microbubble shell surface or more often to a polyethylene glycol (PEG) spacer arm which projects the ligand further away from the microbubble surface. It has been shown that the length of the PEG spacer arm can influence targeting efficiency of microbubbles [74]. For the conjugation of the ligands to the PEG spacer arms, simple biotin-streptavidin linking has been used, whereas covalent bonding with maleimide is also possible. Using both strategies, several thousand ligands can usually be conjugated per square micron of microbubble shell surface [75].

**Figure 4:**
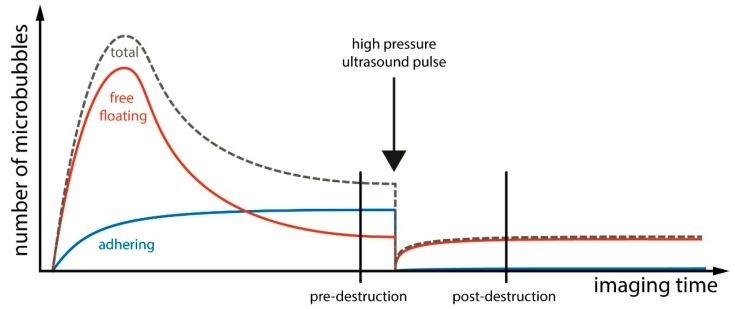
Algorithm for ultrasound molecular imaging. After a bolus injection of targeted microbubbles, a percentage of the total number of microbubbles injected will adhere to the molecule of interest. Freely circulating microbubbles will be cleared in the liver over several minutes. The signal intensity before destruction at the site of interest will be the summation of adhering and free floating microbubbles. The signal intensity after destruction and a short period of reperfusion is generated only by the remaining circulating free microbubbles. Digital subtraction of pre- and post-destruction images can then be used to obtain signal from adhered microbubbles only. Adapted from [76].

Protocols for ultrasound molecular imaging usually involve intravenous bolus injections of several million microbubbles. Over several minutes, microbubbles will adhere to a target of interest within tissue, whereas freely circulating microbubbles are being cleared from the circulation due to shell disintegration and gas loss or clearance in the liver. The presence of attached microbubbles can then be derived from imaging sequences before and after destruction of microbubbles by high-power ultrasound impulses at a timepoint when the total number of freely circulating microbubbles remains nearly constant over a short timespan and is not significantly affected by destruction pulses in the scanplane. On such imaging sequences, the signal before destruction represents the signal generated from the sum of attached and remaining freely circulating microbubbles, whereas, after destruction, only signals from freely circulating microbubbles will be recorded (Figure 4).

**Figure 5:**
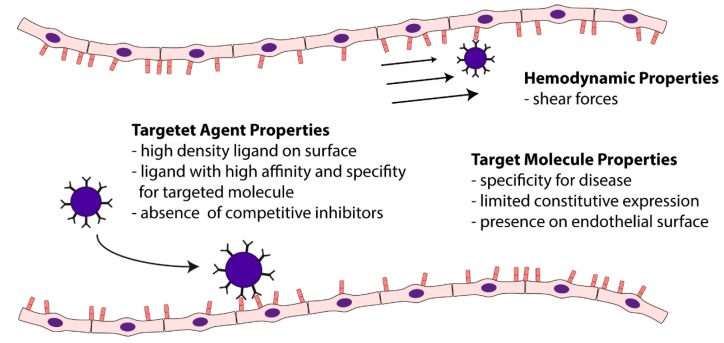
Determinants of targeted micobubble retention. The determinants of targeted microbubble retention within a vessel is dependent on various factors, including the targeted molecules and the contrast agent itself as well as hemodynamic properties. Adapted from [78].

Digital subtraction of signals before and after destruction will then yield the enhancement from attached microbubbles. As microbubbles can be destroyed *in vivo*, repeated injections and imaging sequences, and thus the assessment of several targets in a short timeframe is possible using this technique. However, several factors have to be taken into account for successful ultrasound molecular imaging (Figure 5). The target molecule should be as specific as possible for a particular disease and should possess a limited constitutive expression. As microbubbles are pure intravascular tracers, the molecular target by necessity has to be present on the endothelial surface. The ligand that is used for targeting should be attached in high density to the microbubble surface. It should be specific for the target molecule with the highest possible affinity (fast on-rate, slow off-rate), in particular when used for targeting in high shear conditions such as in large arteries. While, theoretically, microbubble attachment to cells could lead to signal dampening, this has been shown to be minimal *in vitro* [77]. Given these data from *in vitro* studies, sizeable damping of attached microbubbles is unlikely to occur *in vivo*. In this respect, it is important to realize that the subtraction techniques that are commonly employed in ultrasound molecular imaging do not measure the number of adhering microbubbles, but rather the signal generated from the sum of adhering microbubbles, which also depends on other factors for a given number of particles (e.g., attenuation of ultrasound to and from the target particles, size of the particles). Finally, it should be noted that contrast-enhanced ultrasound molecular imaging is in a preclinical stage of development, and no large clinical trials using this technique have been performed to date.

### 5.4. Ultrasound Molecular Imaging of Vascular Inflammation in Atherosclerosis

Inflammation plays an important role in the initiation and progression of atherosclerosis, Leukocyte recruitment to the vessel wall and subsequent transmigration into the underlying tissue is one of the most important contributors to atherosclerosis [79]. Invading monocytes and lymphocytes serve as a source for pro-inflammatory cytokines, reactive oxygen species, pro-angiogenic signals and bioactive products that promote smooth muscle cell migration and further inflammatory cell recruitment as well as pro-thrombotic compounds that have been implicated in the progression of atherosclerosis as well as in complications such as total vessel occlusion in the case of plaque rupture.

Perfluorocarbon-exposed sonicated dextrose albumin (PESDA) microbubbles have been shown to attach to inflamed, dysfunctional endothelium after stretch-induced injury of the carotid arteries during a high fat diet in a pig model [80]. Similarly, increased signal from PESDA microbubbles has been shown in a rat model of early aortic atherosclerosis. The attachment of PESDA microbubbles is complement-mediated, as complement depletion reduced targeted signals [81].

Recruitment of leukocytes to the arterial wall is mediated by cell adhesion molecules that are expressed on the endothelial surface in response to inflammatory stimuli. P-Selectin is responsible for the initial tethering of leukocytes to the activated endothelium by interaction with glycosylated P-Selectin glycoprotein ligand-1 (PSGL-1) [82]. In a mouse model of atherosclerosis, P-Selectin-dependent monocyte rolling has been demonstrated on early atherosclerotic lesions [83] and ultrasound molecular imaging of the expression of P-Selectin has also been validated in the detection of very early atherosclerosis [84]. P-Selectin is, therefore, an important effector in the early inflammatory process and was first successfully used with ultrasound molecular imaging in the detection of renal tissue injury [73] and later in models of myocardial ischemia with the use of sialyl Lewis(x) as a ligand for P-Selectin [85] or anti-P-Selectin antibodies [86].

**Figure 6:**
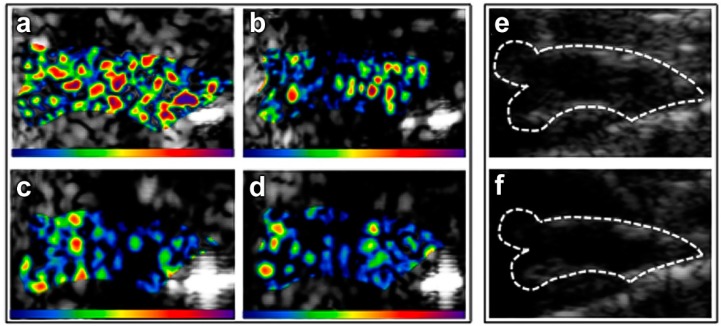
Molecular imaging of the effect of statin treatment on VCAM-1 expression in a mouse model of atherosclerosis. The images show (**a**) high signal in a non-treated animal after injection of VCAM-1 targeted microbubbles; (**b**) low signal in the same mouse after injection of microbubbles bearing a control antibody, whereas in a mouse treated with statins; both VCAM-1 targeted (**c**) and control microbubbles (**d**) show low signal. The color scale for the contrast enhanced ultrasound (CEU) images is shown at the bottom of each frame; (**e**,**f**) illustrate the outline of the ascending aorta on B-mode ultrasound images, which was used as a region of interest for acoustic intensity measurements. Reproduced from [96] with permission.

After initial tethering by P-Selectin, rolling and cytokine-triggered firm adhesion of leukocytes is primarily mediated by endothelial cell adhesion molecules of the immunoglobulin gene superfamily (Vascular cell adhesion molecule 1, VCAM-1 and intercellular adhesion molecule 1, ICAM-1) by interaction with their leukocyte counterligands α4:β1 and αL:β2/αM:β2 [87]. In murine models of atherosclerosis, slow rolling of monocytes on the surface of atherosclerotic plaques appears to be largely mediated by VCAM-1 [88]. With regards to the use as early markers of disease, VCAM-1 and ICAM-1 have been demonstrated to be transcriptionally upregulated on the endothelium of mouse models of atherosclerosis during the very early stages of disease development [89]. Initial *in vitro* studies have confirmed that ICAM-1 expressed on activated human coronary endothelial cells could be targeted using microbubbles [90]. Early *in vivo* studies using echogenic liposomes, targeted to either VCAM-1 or ICAM-1, showed increased signal intensity in yucatan miniswine at sites of injured endothelium, with the drawback of very high intra-arterial doses of liposomes and invasive intravascular ultrasound (IVUS) imaging [91]. VCAM-1 is a prominent target for preclinical contrast-enhanced ultrasound molecular imaging (Figure 6) and was successfully used *in vivo* on ApoE ^−/−^ mice to detect vascular inflammation in established atherosclerotic lesions in the thoracic [92] and abdominal aorta [93].

While the aforementioned studies demonstrated signal enhancement in established atherosclerosis, the most important value of ultrasound molecular imaging is thought to be in the screening of individuals during the very early phases of atherosclerotic disease development. Accordingly, it has been shown that microbubble contrast agents targeted to VCAM-1 or P-Selectin can detect vascular inflammation not only in advanced atherosclerosis but also at lesion-prone sites in murine models of early atherosclerosis [84]. In addition, molecular imaging has been shown to be capable of detecting the effects of anti-oxidative [94,95] or anti-inflammatory [96] treatment on the endothelial phenotype.

### 5.5. Ultrasound Molecular Imaging of Thrombus Formation and Vascular Thrombogenic Potential in Atherosclerosis

In established atherosclerosis, thrombus formation at vulnerable lesions is responsible for complications of atherosclerotic disease such as myocardial infarction or stroke. While thrombus formation within the heart chambers, or even in the aorta, is possible using conventional ultrasound techniques, the detection of smaller thrombi in vessels, such as the coronary arteries, is not possible, and molecular imaging targeted to individual components of thrombi could be of potential value for imaging in small vessels. Unger *et al.* described *in vitro* enhancement of signal from thrombi by targeting microbubbles to activated glycoprotein (GP) IIb/IIIa (integrin α_IIb_:β_3_) on platelets by using a hexapeptide mimicking the carboxyterminal fibrinogen sequence [97]. Similarly, others have used RGD peptide sequences to target activated platelets *in vivo* [98]. Monoclonal antibodies targeting activated GP IIb/IIIa have been used to detect clots and assess the effect of thrombolysis in animal models of carotid artery injury [99,100]. Fusion of antibodies directed against GP IIb/IIIa with fibrinolytic molecules has been successfully used for low-dose targeted thrombolysis in mouse models [101].

While thrombotic complications of atherosclerosis lead to direct clinical complications, platelets-endothelial interactions also play a role in vascular inflammation throughout the pathogenesis of atherosclerosis. P-Selectin, vWF, GP Ibα mediated platelet-endothelial interactions accelerate the formation of atherosclerotic plaques via secretion of cytokines, thus potentiating the vascular inflammation [102,103,104]. Ultrasound molecular imaging has been used in animal models to assess vascular thrombogenicity by targeting activated von Willebrand factor [105]. Coupling of the A1 domain of von Willebrand factor to the microbubble surface has been used in murine atherosclerosis to target GP Ibα on platelets adhering to inflamed endothelium and has been shown to detect responses to short- or long-term therapies aimed at reducing vascular thrombogenicity [94].

## 6. Conclusions

The progress that has been made in the last years in understanding the molecular mechanisms that cause atherosclerosis will lead to novel and improved therapeutic options. Implicit in this is the need for a better *in vivo* assessment of the development of atherosclerosis, and improved risk stratification. Ultrasound imaging is a widely available tool that can assess the morphologic and functional consequences of atherosclerosis. However, it should be noted that while several ultrasound technologies that have recently been developed, such as virtual histology or the contrast based assessment of plaque neovascularization, show promise for risk stratification in specific settings, prospective clinical studies using these techniques are currently lacking. While the aforementioned techniques have already been tested in the clinical arena, ultrasound molecular imaging is still at a preclinical stage, with feasibility proven in a wide array of disease models. However, for translation of these techniques into the clinical field, further technical developments will be necessary. The conjugation strategy using biotin-streptavidin linking of ligands to microbubbles that is widely used in preclinical studies is unlikely to be translatable due to concerns for interference of streptavidin with fatty acid synthesis and gluconeogenesis. As a consequence, biotin-streptavidin conjugation will likely be replaced by covalent bond strategies using amine or sulfhydryl groups. Likewise, antibody-based ligands are likely to be replaced by smaller molecule, lower cost, non-immunogenic ligands. A current limitation of the imaging hardware used for molecular imaging is the inability of ultrasound systems to distinguish attached microbubbles from those that are freely circulating. As a result, this makes the use of post-processing necessary for the detection of signals from targeted microbubbles. However, the steadily increasing computer processing power, which will allow for ultrafast ultrasound imaging in the future, could possibly overcome this limitation and allow for on-line distinction of attached microbubbles from those that are freely circulating [106,107].

Despite these limitations, ultrasound imaging, which is a widely available, relatively easy to use and low-cost non-invasive imaging technique, is well positioned to be of value in risk assessment and diagnoses of atherosclerosis in the future.
